# Glucocorticoid Exposure Induces Preeclampsia *via* DampeningLipoxin A_4_, an Endogenous Anti-Inflammatory and Proresolving Mediator

**DOI:** 10.3389/fphar.2020.01131

**Published:** 2020-07-28

**Authors:** Haojing Liu, Wei Huang, Liping Chen, Qiang Xu, Duyun Ye, Dongxin Zhang

**Affiliations:** ^1^ Department of Science and Education, Wuhan Fourth Hospital, Puai Hospital, Tongji Medical College, Huazhong University of Science and Technology, Wuhan, China; ^2^ Department of Clinical Laboratory, Wuhan First Hospital, Tongji Medical College, Huazhong University of Science and Technology, Wuhan, China; ^3^ Department of Gynecology and Obstetrics, Wuhan Fourth Hospital, Puai Hospital, Tongji Medical College, Huazhong University of Science and Technology, Wuhan, China; ^4^ Department of Pathophysiology, Tongji Medical College, Huazhong University of Science and Technology, Wuhan, China; ^5^ Department of Clinical Laboratory, Wuhan Fourth Hospital, Puai Hospital, Tongji Medical College, Huazhong University of Science and Technology, Wuhan, China

**Keywords:** preeclampsia, glucocorticoid, ALOX5, lipoxin A_4_, leukotriene B_4_

## Abstract

The pathogenesis of preeclampsia (PE) involves several pathophysiological processes that may be affected by glucocorticoid (GC). We confirmed previously that GC exposure could result in PE, while PE is linked to a deficiency of lipoxin A_4_ (LXA_4_), an endogenous dual anti-inflammatory and proresolving mediator. The present study was to investigate whether GC exposure induces PE *via* dampening LXA_4_. In the study, cortisol levels of PE women were higher than those of normal pregnancies, LXA_4_ levels were downregulated in both PE patients and GC-mediated PE rats, and leukotriene B_4_ (LTB_4_) levels were upregulated in both PE patients and GC- mediated PE rats. Moreover, cortisol levels were negatively correlated to LXA_4_ levels, while positively correlated to LTB_4_ levels in PE patients. Mechanically, GC downregulated LXA_4_ *via* disturbing its biosynthetic enzymes, including ALOX15, ALOX5B and ALOX5, especially activating ALOX5, the key enzyme for class switching between LXA_4_ and LTB_4_. Importantly, replenishing LXA_4_ could ameliorate PE-related symptoms and placental oxidative stress in PE rat model induced by GC. Moreover, LXA_4_ could inhibit GC-mediated ALOX5 activation and LTB_4_ increase, and also suppress 11β-HSD2 expression and corticosterone upregulation. The protective actions of LXA_4_ might be explained by its roles in antagonizing the adverse effects of GC on trophoblast development. Together, our findings indicate that GC exposure could contribute to PE through dampening LXA_4_, and GC/LXA_4_ axis may represent a common pathway through which PE occurs.

## Introduction

Preeclampsia (PE), characterized by maternal hypertension, proteinuria, and other systemic disorders occurring after 20 weeks of gestation, remains a leading cause of maternal and fetal morbidity and mortality (Noris et al., 2005; Redman and Sargent, 2005; Young et al., 2010). Upon pregnancy, placenta might be exposed to glucocorticoid (GC) through several ways, including GC metabolism damage, synthetic GC administration and maternal stress (Michael and Papageorghiou, 2008; Xu et al., 2013). Although the pathogenesis of PE remains largely elusive, increasing evidence indicates that PE is associated with stress or GC exposure (Klonoff-Cohen et al., 1996; Kurki et al., 2000; László et al., 2013; Xu et al., 2014; Zhang et al., 2016). A previous report demonstrated that stress in early pregnancy might lead to an increase in the risk of early-onset PE (László et al., 2013). Moreover, another report showed that stress in early pregnancy could contribute to PE in rat (Takiuti et al., 2002). Interestingly, we confirmed recently that GC exposure in early placentation could induce PE in rats (Zhang et al., 2016; Zhang et al., 2018).

PE is a systemic inflammatory condition in which oxidative stress and endothelial dysfunction occurs (Das, 2015). Cotechini et al. showed that inflammation in rat pregnancy inhibits spiral artery remodeling leading to PE-related features and fetal growth restriction (Cotechini et al., 2014), providing the rationale for the use of anti-inflammatory mediators in the prevention and treatment of inflammation-associated PE. It is proposed that PE may occur due to deficiency of polyunsaturated fatty acids (PUFAs) and their anti-inflammatory products specialized pro-resolving mediators (SPMs), including lipoxins, resolvins, protectins, and maresins (Das, 2015). As one of the most important endogenous SPMs, Llipoxin A_4_ (LXA_4_), derived from arachidonic acid (AA) involving the dual lipoxygenation of arachidonic acid enzymatically by lipoxygenases (LOXs), function as ‘‘stop signal’’ in inflammation (Serhan et al., 2008). It has been demonstrated that LXA_4_ levels are greater in pregnant compared with nonpregnant women, with a progressive increase across pregnancy (Maldonado-Pérez et al., 2011), and LXA_4_ is a novel estrogen receptor modulator (Russell et al., 2011). Together with our findings, increasing studies indicate that LXA_4_ exerts vital roles in homoeostasis restoration and inflammation termination of reproductive system (Maldonado-Pérez et al., 2011; Hutchinson et al., 2011; Macdonald et al., 2011; Lin et al., 2012; Xu et al., 2014; Zhang et al., 2014; Liu et al., 2020). In fact, we found that PE is linked to a deficiency of LXA_4_ that could improve PE-related symptoms in rat model (Lin et al., 2012; Xu et al., 2014). Moreover, a previous study by Villa et al. demonstrated the protective roles of aspirin triggered-LXA_4_ (ATL, 15-epi-LXA_4_) on PE-related inflammation (Gil-Villa et al., 2012). Thus, GC exposure-mediated PE may involve the loss of LXA_4_.

The present study was undertaken to confirm the involvement of GC/LXA_4_ axis in PE pathogenesis and to reveal the underlying mechanisms. We examined whether GC-mediated PE is explained by the dysregulation of LXA_4_ biosynthesis. The modulation of LXA_4_ on Dex-mediated PE was also investigated. The study would provide an important clue for better understanding of PE pathogenesis and contribute to develop potential therapeutic strategies to prevent PE.

## Materials and Methods

### Reagents and Antibodies

Dex sodium phosphate, Dex, 3-[4,5-dimethylthiazol-2-yl]-2,5-diphenyltetrazolium bromide (MTT), and Hoechst 33258 were purchased from Sigma Aldrich (Allentown, PA, USA). LXA_4_ solution (Cayman Chemical, Ann Arbor, MI, USA) was preserved at -80°C until diluted in cell culture medium or sterile saline immediately before use. Anti-HIF1A, -ALOX15, -ALOX15B, -β-actin, and -Lamin B antibodies were from Santa Cruz Biotechnology (Santa Cruz, CA, USA). Mouse anti-ALOX5 monoclonal antibody was from BD Biosciences. Anti-11β-HSD2 antibody was from Biosynthesis Biotechnology Company (Beijing, China). RIPA Lysis Buffer and Nuclear and Cytoplasmic Protein Extraction Kit were from Beyotime Biotechnology (Shanghai, China). BCA Protein Assay Kit was from Pierce (Rockford, IL, USA).

### Patient Samples

Blood specimens were from 26 pregnant women: 13 pregnant women with PE defined by proteinuria (0.3 g/day) and hypertension (systolic and diastolic blood pressures over 140/90 mmHg) (Higgins and de Swiet, 2001) were recruited from Wuhan Fourth Hospital (Puai Hospital, Tongji Medical College, Huazhong University of Science and Technology); as control group, 13 women were selected with characteristics similar to those presented by PE patients, including gestational age, body mass index, eliminated kidney disease, high blood pressure, diabetes, and so on. Blood and placenta specimens were from diagnosed patients after informed consent and approved by the Medical Ethical Committee of Tongji Medical College, Huazhong University of Science and Technology in accordance with Declaration of Helsinki.

The patients didn’t receive any medications before samplings collection. Blood extracted from pregnant women stood for 20 min at room temperature, and was then centrifuged at 4,000 rpm for 5 min for obtaining serum. The serum was stored at -80°C for further detection.

### Animals and Experimental Protocols

Female Sprague-Dawley rats (10–12 weeks old, weighing 220–250 g) were from the Experimental Animal Center of Tongji Medical College, Huazhong University of Science and Technology. Animals were housed individually in plastic cages with wood chips as bedding under pathogen-free conditions, in a controlled environment of temperature at 20–25°C and 12 h cycles of light and dark. Rats were fed a standard laboratory diet and water *ad libitum*. Pregnancy was obtained by mating female rats with fertile male rats at a ratio of 2:1 overnight. Daily vaginal smears were observed, and appearance of spermatozoa in vaginal smear was defined as gestational day (GD) 1. All animal works were conducted according to the Guide for the Care and Use of Laboratory Animals of the National Institutes of Health. All studies involving rats were approved by Animal Care and Use Committee of Huazhong University of Science and Technology.

#### Experimental Protocol 1

Pregnant rats were randomly divided into control group and treatment group. The rats in treatment group were injected subcutaneously (*s.c.*) with Dex sodium phosphate (2.5 mg/kg per day) from GD7 to 13, and control rats were injected *s.c.* with equal saline. On GD21, rats were fully anesthetized with chloral hydrate and placental samples were rapidly extracted. Blood specimens were drawn by heart puncture and centrifuged at 3,000 rpm for 20 min at 4°C to collect the serum.

#### Experimental Protocol 2

Pregnant rats were randomly divided into Control group, Dex group and Dex+LXA_4_ group. From GD7 to GD13, control rats were injected *s.c.* with equal saline, rats in Dex group were injected *s.c.* with Dex sodium phosphate (2.5 mg/kg per day), and rats in Dex+LXA_4_ group were injected with Dex sodium phosphate (2.5 mg/kg per day) *s.c.* plus LXA_4_ (10 μg/kg per day) intraperitoneally (*i.p.*). On GD21, rats were fully anesthetized with chloral hydrate and the uterus was removed and placed in a chilled dish. Placenta and pup were rapidly extracted. All of the pups were weighed, and litter size noted. All samples were analyzed individually.

### Measurement of Systolic Blood Pressure (SBP)

At indicated time (initial non-pregnant status, GD3–5, GD14, and GD20), SBP was detected in conscious and restrained pregnant rats. An automated system with a photoelectric sensor linked to a dual channel recorder (BP-98A, Softron, Japan), tail cuff and sphygmomanometer was used to obtain blood pressure measurements, which have been previously demonstrated to be closely correlated with arterial measurements (Mulvany and Halpern, 1977). The measurements were repeated three times for each rat, with the mean value recorded.

### Determination of Urinary Albumin Excretion

On GD5 and GD20, the pregnant rats were placed in metabolic cages for collecting 24 h urine. To avoid contamination of the collected urine, rats were restricted from food; however, they were allowed free access to water. To avoid the adverse effects of fasting, rats were fed in other cages for 30 min every 6 h. Urine samples were centrifuged at 3,000 rpm for 20 min at room temperature, and the supernatant was collected for urinary albumin analysis. Urine protein concentrations were detected by using pyrogallol red in an automatic biochemical analyzer (ADVIA 2400 Chemistry System, Siemens Medical Solutions Diagnostics, NY, USA).

### Measurement of Thiobarbituric Acid Reactive Substances (TBARS)

TBARS in placenta was determined by employing a commercially available kit (QuantiChrom™ TBARS Assay Kit, DTBA-100) according to the manufacturer’s instructions (BioAssay Systems, USA). Briefly, placental tissues (20 mg or so) were placed into 200 μl ice-cold phosphate buffered saline with protease inhibitors. The tissues were first homogenized thoroughly and then sonicated for 20 s on ice. The homogenates were then centrifuged at 3,000 rpm for 10 min at 4°C. 20 μl aliquot was used for protein determination. The resultant absorbances were read at 535 nm. TBARS levels were expressed as nmol/mg protein.

### Cell Culture and Treatment

Human first-trimester trophoblast cell line HTR-8/SVneo were from American Type Culture Collection (USA). Cells were cultured at 37°C in a humidified atmosphere with 5% CO_2_ in 1,640 medium supplemented with 10% heat-inactivated fetal bovine serum, 25 mM HEPES, 100 U/ml penicillin G and 100 U/ml streptomycin. Cells were treated with Dex (10 (^-7^) mol/L) or Dex (10^-7^ mol/L) plus LXA_4_ (100 nM) for cell proliferation assay, and Dex (10^-6^ mol/L) or Dex (10^-6^ mol/L) plus LXA_4_ (100 nM) for cell apoptosis assay.

### Cell Proliferation and Apoptosis Assay

Cell proliferation was measured by a colorimetric method based upon metabolic reduction of the soluble yellow tetrazolium dye MTT to its insoluble purple formazan. Approximately 5,000 cells/well were grown in 96-well plates and incubated overnight in 200 μl of cell culture medium. After cells were treated with indicated conditions for 24 h, each well was added with

20 μl MTT (0.5 mg/ml) and incubated for 4 h before supernatant was removed. After plate was placed at 37°C for 15 min in 150 μl DMSO, the absorbency was detected with a micro ELISA reader (Amersham Biosciences, USA) at 492 nm.

Cell apoptosis was determined by Hoechst staining. Cells were incubated with Hoechst dye for 15 min before examination under fluorescence microscope. Apoptotic cells were characterized by characteristic nuclear fragmentation; only those nuclei showing evidence of DNA fragmentation without plasma membrane damage were determined to be apoptotic cells.

### Western Blotting

Total protein and nuclear protein of placental tissue were extracted by using RIPA Lysis Buffer and Nuclear and Cytoplasmic Protein Extraction Kit respectively for assessing different protein expression. Protein concentrations were detected by using BCA Protein Assay Kit. Then, regular western blotting assay was performed. Briefly, equal amounts of protein were subjected to 12% SDS-PAGE electrophoresis and transferred to PVDF membranes (Millipore, USA). The membranes were blocked with 5% (w/v) non-fat dried milk and incubated with different primary antibodies overnight at 4°C, followed by incubation with a HRP-conjugated secondary antibody. The bound antibody was detected by an enhanced chemiluminescence kit (Millipore, USA) on X-ray film. Lamin B and β-actin served as internal control of nuclear protein and total protein respectively.

### Measurement of Cortisol, LXA_4_, Leukotriene B4 (LTB_4_), and Corticosterone

Before measurement of cortisol, LXA_4_, LTB_4_, and corticosterone levels in placenta, tissue homogenization was firstly performed to extract the placental tissue contents. Briefly, placental samplings were homogenized in mild homogenate buffer containing a cocktail enzyme inhibitor by using glass homogenizer. 20 min later, the homogenates were centrifuged at 3,000 rpm at 4°C for 20 min. Supernatants were collected and stored at -80°C until detection. The levels of cortisol in serum and placenta were detected by enzyme chemiluminescence immunoassay in chemiluminescence analyzer (ADVIA Centaur XP Immunoassay System, Siemens Healthcare Diagnostics Inc., NY, USA). The levels of LXA_4_ and LTB_4_ in serum and placenta were determined by assay kit according to the manufacturers’ protocols (Neogen Corporation, Lansing, MI, USA; Cayman Chemical Company, USA) respectively. Corticosterone in rat placenta was quantified using a commercial ELISA kit for corticosterone (R&D Systems, Minneapolis, MN, USA) according to the manufacturer’s instructions.

### Statistical Analysis

All statistical analyses were performed by using the SPSS 19.0 software. The results were expressed as means ± S.E.M of multiple independent experiments. The means of different groups were compared by employing either Student’s t-test or one-way ANOVA followed by S-N-K post-hoc test. A value of p < 0.05 was considered significant.

## Results

### The Expressions of Cortisol and LXA_4_ in PE Patients

**Figure 1:**
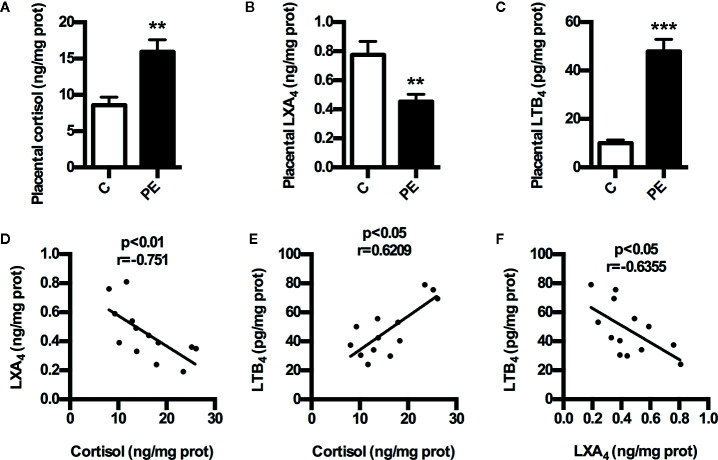
Expressions of cortisol and LXA_4_ in human PE. **(A)** Comparison of placental cortisol levels between PE patients and healthy controls; **(B)** Comparison of placental LXA_4_ levels between PE patients and healthy controls; **(C)** Comparison of placental LTB_4_ levels between PE patients and healthy controls; **(D–F)** The correlation between cortisol, LXA_4_ and LTB_4_ in PE patients. Results are expressed as means ± SEM (n=13 in each group). **p < 0.01 and ***p < 0.001 versus control group, two-tailed Student’s t-test.

To determine whether GC is correlated with LXA_4_ in PE, we detected the expressions of cortisol and LXA_4_ in placenta of both PE patients and normal pregnancies. As shown in **Figures 1A, B**, the levels of placental cortisol in PE patients showed significant increase compared to normal pregnancies,while placental LXA_4_ levels of PE patients decreased when compared to normal pregnancies. Meanwhile, placental LTB_4_ levels of PE patients were higher than those of normal pregnancies (**Figure 1C**). In line with this, serum LXA_4_ levels of PE patients were lower than those of normal pregnancies and the levels of serum LTB_4_ in PE patients showed increase compared to normal pregnancies, although the levels of serum cortisol in PE patients showed non-significant increase compared to normal pregnancies (**Supplementary Figure 1**). Importantly, correlation analysis showed that there was significant negative correlation between cortisol and LXA_4_, positive correlation between cortisol and LTB_4_, and negative correlation between LXA_4_ and LTB_4_ (**Figures 1D–F**). Mechanistically, we have confirmed abnormal expressions of LXA_4_ biosynthesis enzymes during PE that both ALOX15 and ALOX15B are downregulated, while ALOX5 is activated in PE patients (Liu et al., 2020). Together, these clinical data suggest that LXA_4_ deficiency is associated with GC exposure in human PE.

### GC Exposure Inhibits LXA_4_ Biosynthesis in PE Rats

**Figure 2:**
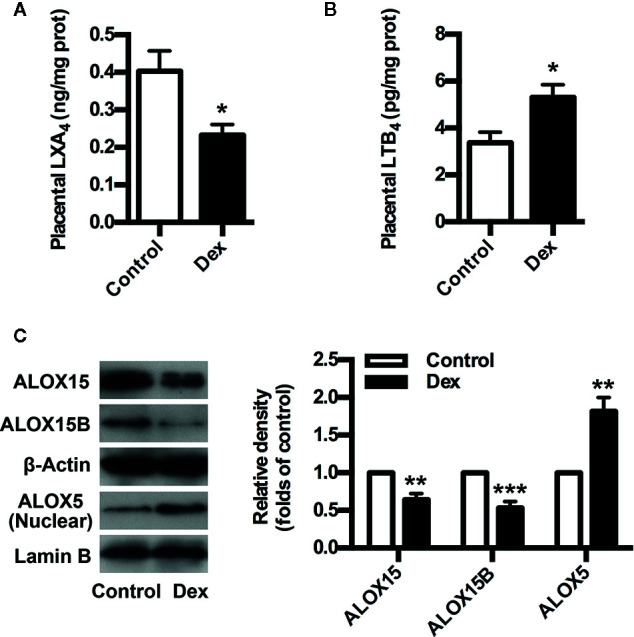
GC inhibits LXA_4_ biosynthesis in experimental PE rats. Pregnant rats were treated according to *Experimental protocol 1*. **(A)** Effect of Dex on placental LXA_4_ levels in pregnant rats; **(B)** Effect of Dex on placental LTB_4_ levels in pregnant rats; **(C)** Effect of Dex on ALOX15, ALOX15B, and ALOX5 protein expression in placenta of pregnant rats. Protein expression was detected by western blotting. The histogram represents means ± SEM of the densitometric scans for protein bands (n=7 rats in each group), normalized by comparison with β-Actin and Lamin B and expressed as a percentage of Control. Results are expressed as means ± SEM (n=7 rats in each group). *p < 0.05, **p < 0.01, and ***p < 0.001 versus control group, two-tailed Student’s t-test.

To confirm the involvement of GC/LXA_4_ axis in PE pathogenesis, an GC-mediated PE rat model was constructed as previously (Zhang et al., 2018), and the levels of LXA_4_ in placenta of experimental PE rats were detected. As shown in **Figure 2A**, placental LXA_4_ was strikingly down-regulated by Dex in experimental PE rats, while placental LTB_4_ levels of PE rats were up-regulated by Dex (**Figure 2B**). To determine the mechanisms underlying the effects of GC on LXA_4_ production, we detected the expressions of ALOX15, ALOX15B and ALOX5 in placenta of experimental PE rats. Dex treatment contributed to the downregulation of both ALOX15 and ALOX15B while the enhancement of ALOX5 nuclear translocation (**Figure 2C**), suggesting GC-mediated disturbance of biosynthesis enzymes may be the reason for LXA_4_ deficiency in PE. These results indicate that GC exposure may lead to PE through dampening LXA_4_ biosynthesis.

### LXA_4_ Improves PE-Related Symptoms and Placental Oxidative Stress of PE Rats

**Figure 3:**
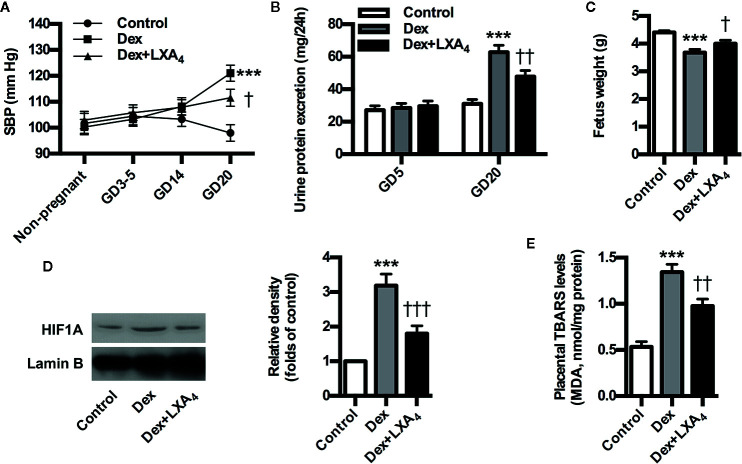
LXA_4_ ameliorates PE-related symptoms and placental oxidative stress of GC-mediated rat PE. Pregnant rats were treated according to *Experimental protocol 2*. Systolic blood pressure (SBP), 24 h urinary protein excretion and fetal weight are presented in **(A–C)** respectively; **(D)** Effect of LXA_4_ on Dex-mediated placental HIF1A expression. HIF1A was detected by western blotting. The histogram represents means ± SEM of the densitometric scans for protein bands (n=7 rats in each group), normalized by comparison with Lamin B and expressed as a percentage of Control; **(E)** Effect of LXA_4_ on Dex-mediated placental lipid peroxidation. Levels of lipid peroxidation were determined by using a commercially available TBARS kit. The measurements of lipid peroxidation represent the degree of placental oxidative stress. Results are expressed as means ± SEM (n=7 rats in each group). ***p < 0.001 versus control group, ^†^P < 0.05, ^††^P < 0.01 and ^†††^P < 0.001 versus Dex group, one-way ANOVA with S-N-K posttest.

To further validate the involvement of GC/LXA_4_ axis in the pathogenesis of PE, the experimental PE rats were treated with LXA_4_. As shown in **Figure 3A**, SBP was elevated after Dex exposure, while blood pressure conditions were improved after LXA_4_ administration. Dex exposure increased the urine protein level on GD20, while LXA_4_ could alleviate the urine protein excretion (**Figure 3B**). LXA_4_ could also ameliorate intrauterine growth restriction as showed by the fetal rat weight (**Figure 3C**) (**Figure 3D**). To clarify the effects of LXA_4_ on placental oxidative stress, placental oxidative damage and HIF1A expression were determined by using TBARS assay kit and western blotting respectively. As shown in **Figure 3D, E**, HIF1A was overexpressed in placenta of experimental PE rats, and Dex exposure contributed to increased TBARS levels in placenta, suggesting placental oxidative impairment. But after LXA_4_ treatment, both TBARS levels and HIF1A expressions were ameliorated (**Figures 3D, E**). These results suggest that LXA_4_ is effective in relieving PE-related symptoms and placental oxidative stress of experimental PE rats induced by GC.

### LXA_4_ Suppresses GC-Mediated ALOX5 Activation and LTB_4_ Upregulation in Experimental PE Rats

**Figure 4:**
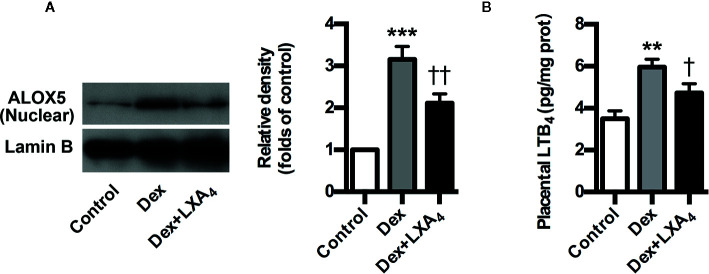
LXA_4_ inhibits GC-mediated ALOX5 activation and LTB_4_ production in experimental PE rats. Pregnant rats were treated according to *Experimental protocol 2*. **(A)** Effect of LXA_4_ on Dex-mediated placental ALOX5 nuclear translocation. ALOX5 nuclear translocation was detected by western blotting. The histogram represents means ± SEM of the densitometric scans for protein bands (n=7 rats in each group), normalized by comparison with Lamin B and expressed as a percentage of Control; **(B)** Effect of LXA_4_ on Dex-mediated placental LTB_4_ increase. Results are expressed as means ± SEM (n=7 rats in each group). **p < 0.01 and ***p < 0.001 versus control group, ^†^P < 0.05 and ^††^P < 0.01 versus Dex group, one-way ANOVA with S-N-K posttest.

ALOX5 is the key enzyme for the biosynthesis of both lipoxins (LXs) and leukotrienes (LTs). Upon stimulation, it translocates onto the nuclear membrane to form active enzyme complex that converts AA to LTs (Soberman and Christmas, 2003). ALOX5 activation indicates possible class switching from LXs to LTs production that was confirmed by our present results that Dex-mediated ALOX5 activation leaded to increased LTB_4_ but decreased LXA_4_ production in placenta of experimental PE rats (**Figures 4A, B**). However, LXA_4_ treatment could suppress Dex-induced ALOX5 nuclear translocation and LTB_4_ increase in placenta of experimental PE rats (**Figures 4A, B**). These data indicate that LXA_4_ could inhibit GC-mediated ALOX5 activation and LTB_4_ upregulation in placenta of experimental PE rats.

### LXA_4_ Is Involved in 11β-HSD2 Upregulation in Experimental PE Rats

**Figure 5:**
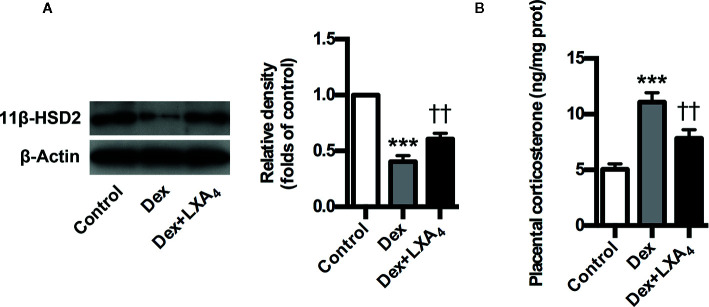
LXA_4_ suppresses GC-mediated 11β-HSD2 expression and corticosterone production in experimental PE rats. Pregnant rats were treated according to *Experimental protocol 2*. **(A)** Effect of LXA_4_ on Dex-mediated placental 11β-HSD2 expression. 11β-HSD2 expression was detected by western blotting. The histogram represents means ± SEM of the densitometric scans for protein bands (n=7 rats in each group), normalized by comparison with β-Actin and expressed as a percentage of Control; **(B)** Effect of LXA_4_ on Dex-mediated placental corticosterone increase. Results are expressed as means ± SEM (n=7 rats in each group). ***p < 0.001 versus control group, ^††^P < 0.01 versus Dex group, one-way ANOVA with S-N-K posttest.

Given that 11β-HSD2 has a well recognized function as a potent dehydrogenase that rapidly inactivates GC, and is a key enzyme for controlling local GC activity in placenta (Seckl and Walker, 2001; Tomlinson et al., 2007; Causevic and Mohaupt, 2007), we determined placental 11β-HSD2 changes and the modulation of LXA_4_ on 11β-HSD2 in experimental PE rats. Placental 11β-HSD2 was strikingly decreased by Dex exposure in experimental PE rats, as evaluated by western blotting (**Figure 5A**). In line with this, placental corticosterone levels of PE rats were higher than those of controls (**Figure 5B**). Surprisingly, LXA_4_ could antagonizes the effects of Dex exposure on 11β-HSD2 expressions (**Figures 5A, B**). Therefore, 11β-HSD2 downregulation in placenta might contribute to increases in local GC activity, and LXA_4_ may be involved in the up-regulation of 11β-HSD2 in PE.

### LXA_4_ Antagonizes the Effects of GC on Trophoblast Proliferation and Apoptosis

**Figure 6:**
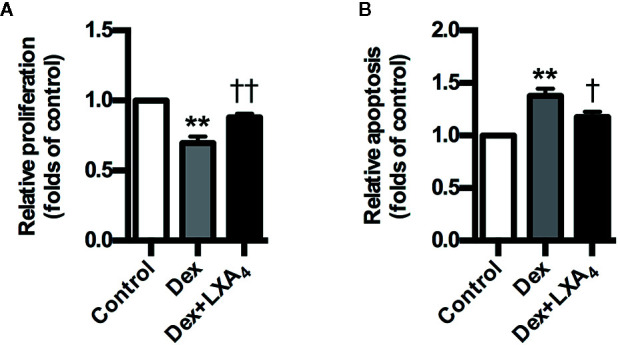
LXA_4_ antagonizes the effects of GC on trophoblast proliferation and apoptosis *in vitro*. HTR-8/SVneo cells were treated with Dex (10^-7^ mol/L) or Dex (10^-7^ mol/L) plus LXA_4_ (100 nM) for 24 h in proliferation assay **(A)** and treated with Dex (10^-6^ mol/L) or Dex (10^-6^ mol/L) plus LXA_4_ (100 nM) for 24 h in apoptosis assay **(B)**. **(A)** LXA_4_ reverses the inhibitory effect of Dex on HTR-8/SVneo proliferation; **(B)** LXA_4_ inhibits the promotion effect of Dex on HTR-8/SVneo apoptosis. Results are expressed as means ± SEM from three independent experiments. **P < 0.01 versus control group, ^†^P < 0.05 and ^††^P < 0.01 versus Dex group, one-way ANOVA with S-N-K posttest.

Dex suppressed the proliferation of trophoblast cell line HTR-8/SVneo, while it stimulated HTR-8/SVneo apoptosis *in vitro* (**Figures 6A, B**). However, LXA_4_ treatment could antagonize the effects of Dex on HTR-8/SVneo proliferation and apoptosis (**Figures 6A, B**). These results suggest that LXA_4_ could improve the adverse effects of Dex on trophoblast development *in vitro*, supporting the protective roles of LXA_4_ on trophoblast against GC.

## Discussion

Up to now, the molecular mechanisms underlying the pathogenesis PE remain elusive. The study provides evidence that GC exposure might lead to PE through dampening LXA_4_, an endogenous protective anti-inflammatory and proresolving mediator during pregnancy, supporting that GC/LXA_4_ axis might represent a common pathway through which PE occurs. Meanwhile, our data demonstrate the mechanisms underlying the modulation of LXA_4_ on GC in PE.

The study revealed several novel findings regarding the relationship between GC and LXA_4_ in PE. Foremost, the study reports that LXA_4_ deficiency is associated with GC exposure in human PE, thus providing the clinical data of involvement of GC/LXA_4_ in PE. Furthermore, the experimental PE rat model confirms that GC exposure could result in PE through dampening LXA_4_ biosynthesis, while LXA_4_ could improve PE-related symptoms and placental oxidative stress of experimental PE rats. Lastly, LXA_4_ could inhibit GC-mediated ALOX5 activation and LTB_4_ upregulation in experimental PE rats, and antagonize the effects of GC on11β-HSD2 expression and trophoblast development. Thus, the study is the first to demonstrate that GC exposure induces PE *via* dampening LXA_4_, supporting GC/LXA_4_ axis as a common pathway through which PE occurs.

Increasing evidence indicate the adverse effects of stress or GC exposure on pregnancy (Michael and Papageorghiou, 2008; Xu et al., 2013; László et al., 2013; Lin et al., 2013). Previous reports showed that stress could induce PE in pregnant women and rats (Takiuti et al., 2002; László et al., 2013). We confirmed recently that GC exposure in early pregnancy could result in PE-related symptoms in pregnant rats (Zhang et al., 2016; Zhang et al., 2018), indicating GC exposure as a potential stimulus for PE development. In the study, pregnant rats exposed to GC were characterized by decreased LXA_4_ expression, suggesting these PE-related manifestations have occurred, at least in part, in response to LXA_4_ down-regulation mediated by GC since LXA_4_ is an endogenous protective anti-inflammatory and proresolving mediator during pregnancy. In fact, cortisol levels were negatively correlated to LXA_4_ levels in PE patients, and LXA_4_ biosynthesis were dampened by GC in PE rats.

LXA_4_ biosynthesis is controlled by several enzymes through transcellular process, enzymatically by LOXs involving the dual lipoxygenation of AA (Serhan et al., 2008). Recently, we studied the expressions of LXA_4_-biosynthesis enzymes ALOX15, ALOX15B and ALOX5 in PE women, and confirmed the downregulation of ALOX15B and ALOX15 while the activation of ALOX5 (Liu et al., 2020). ALOX5 is the key enzyme for the biosynthesis of both LTs and LXs. Upon stimulation, it translocates onto the nuclear membrane to form active enzyme complex that converts AA to LTs (Soberman and Christmas, 2003). ALOX5 activation indicates possible class switching from LXs to LTs synthesis, which has been confirmed by our previous work that ALOX5 activation resulted in increased LTB_4_ but reduced LXA_4_ production (Zhang et al., 2014; Liu et al., 2020). Here, we showed LXA_4_ deficiency and LTB_4_ increase in PE patients and GC-mediated experimental PE rats. Importantly, we demonstrated negative association between LXA_4_ and GC in PE patients. By employing PE rat model, we demonstrated that GC exposure could inhibit LXA_4_ production *via* downregulating ALOX15 and ALOX15B while activating ALOX5. Thus, GC-mediated inhibition of ALOX15B and ALOX15 while activation of ALOX5 was responsible for abnormal LXA_4_ biosynthesis during PE, providing the evidence supporting the involvement of GC/LXA_4_ axis in PE pathogenesis. We next elucidated the mechanisms determining the counterregulatory effects of LXA_4_ on GC. We found that LXA_4_ could inhibit GC-mediated ALOX5 activation and LTB_4_ upregulation in experimental PE rats. The present study has demonstrated an additional function of LXA_4_ to alleviate the adverse effects of GC during pregnancy by inhibiting ALOX5 activation.

In the study, Dex exposure resulted in proteinuria, hypertension and restricted fetus, all classic hallmarks of PE. Interestingly, LXA_4_ supplementation significantly improved PE-related symptoms induced by Dex, suggesting the protective effects of LXA_4_ on GC-mediated PE. Furthermore, LXA_4_ treatment improved placental oxidative stress in PE rat mediated by Dex. Placenta oxidative stress represents the fundamental abnormality resulting in PE (Redman and Sargent, 2000). We confirmed here deduced placental oxidative stress after LXA_4_ administration in Dex-induced PE rat model that supports the protective roles of LXA_4_ against adverse effects of GC on placental function. HIF1A, overexpressed in hypoxic PE placenta, is implicated in PE pathogenesis (Noris et al., 2005; Young et al., 2010; Tal, 2012). Importantly, we reported that LXA_4_ could suppress HIF1A overexpression in Dex-treated placenta, providing an important mechanistic explanation for the modulation of LXA_4_ on GC in PE.

The human placenta express high levels of 11β-HSD2, the enzyme responsible for GC inactivation (Seckl and Walker, 2001; Tomlinson et al., 2007; Causevic and Mohaupt, 2007). It has been suggested that 11β-HSD2 serves as an effective barrier to protect the placenta and fetus from high concentrations of maternal cortisol (Causevic and Mohaupt, 2007). Pregnancies complicated by PE have been associated to a compromised 11β-HSD2 activity and/or availability (Causevic and Mohaupt, 2007). We found here that Dex exposure could lead to enhanced 11β-HSD2 expression and corticosterone production in experimental PE rats. However, LXA_4_ could antagonize the effects of GC on11β-HSD2 expression, and, accordingly, inhibit corticosterone production in experimental PE rats. The study demonstrated an additional function of LXA_4_ to inhibit the adverse effects of GC during pregnancy by suppressing 11β-HSD2 expression and GC activity.

PE is attributed to impaired trophoblast development, called poor placentation (Redman and Sargent, 2005; Fisher, 2015). We confirmed previously that GC exposure in early pregnancy could result in PE in rats *via* inhibiting trophoblast development (Zhang et al., 2016). We showed here that Dex could promote trophoblast apoptosis while suppress trophoblast proliferation. However, LXA_4_ could antagonize the effects of Dex on trophoblast apoptosis and proliferation. Our results suggest that LXA_4_ could ameliorate the inhibitory effects of Dex on trophoblast development, supporting the protective roles of LXA_4_ in placentation against GC and providing another important mechanistic explanation for the counterregulation of LXA_4_ on GC in PE.

PE, involving complicated mechanisms in its pathogenesis, is thought to be a multifactorial disorder. Thus, the study just demonstrates one of the important mechanisms underlying PE occurrence. It is noteworthy that there may be other factors, including reduction of PPAR activators, imbalance of angiogenic and anti-angiogenic factors, enhancement of pro-inflammatory cytokines, abnormal expressions of microRNAs, and impairment of other SPMs (resolvins, protectins and maresins) (Waite et al., 2005; Das, 2015; Das, 2018), in addition to dampened LXA_4_ that are responsible for GC-mediated PE, and may act in concert to stimulate HIF1A overexpression, placental oxidative stress and PE development reported in the study and other works. Further studies are planned to investigate other detailed mechanisms underlying the regulation of LXA_4_ and GC on these factors during PE. In addition, further studies may be needed to explore other candidate agents, such as statins and placental growth factor (PGF), for enhancing LXA_4_ biosynthesis and PE treatment, since pravastatin has been demonstrated to induce PGF and ameliorate PE in a mouse model (Kumasawa et al., 2011).

By employing clinical data, the present study demonstrated the correlation between GC and LXA_4_ in human PE. Relying on the findings gathered by employing GC-induced PE rat model and trophoblast model, the study provides further evidence that GC/LXA_4_ axis is involved in PE pathogenesis. The study demonstrates a common mechanism underlying PE occurrence, providing new insights into the better understanding of PE. Our data reveal a previously unappreciated facet of LXA_4_ biofunctions, implicating the endogenous lipid mediator in novel immunoendocrine crosstalk mechanisms during PE. These findings suggest that administration of LXA_4_ and avoidance of stress or GC exposure might represent potential strategies to prevent PE, and also contribute to develop other therapeutic strategies to treat PE.

## Data Availability Statement

All datasets presented in this study are included in the article/**Supplementary Material**.

## Ethics Statement

The studies involving human participants were reviewed and approved by Ethical Committee of the Medical Faculty of Tongji Medical College, Huazhong University of Science and Technology. The patients/participants provided their written informed consent to participate in this study. The animal study was reviewed and approved by Animal Care and Use Committee of Huazhong University of Science and Technology.

## Author Contributions

DZ conceived the project. HL, WH, LC, and QX performed the experiments. HL, WH, DZ, and DY analyzed the results. DZ and DY wrote the manuscript.

## Funding

This work was supported by the National Natural Science Foundation of China [grant numbers 81701469, 81671480, 81903217]; the Application foundation frontier project of Wuhan Science and Technology Bureau; the Young and Mid-Aged Key Medical Personnel Training Project of Wuhan City; the Young Key Talent Project of Wuhan Fourth Hospital.

## Conflict of Interest

The authors declare that the research was conducted in the absence of any commercial or financial relationships that could be construed as a potential conflict of interest.
