# Supplementary material for: Glucocorticoid Exposure Induces Preeclampsia via DampeningLipoxin A4, an Endogenous Anti-Inflammatory and Proresolving Mediator
Source: Front Pharmacol. 2020 Jul 28;11:1131. doi: 10.3389/fphar.2020.01131 (PMC7399346; doi:10.3389/fphar.2020.01131)
Supplement: Supplementary file 1 [file Image_1.pdf]

Supplementary Figure

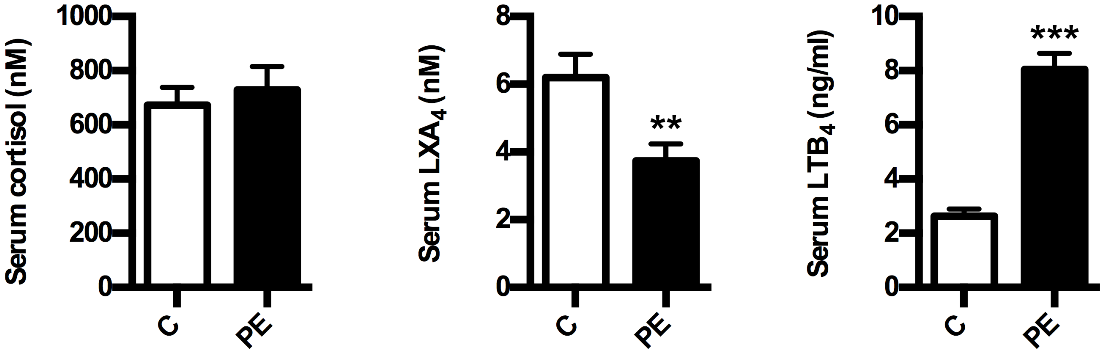

Supplementary Figure 1. Serum levels of cortisol and LXA_4 in human PE. (A) Comparison of serum cortisol levels between PE patients and healthy controls; (B) Comparison of serum LXA_4 levels between PE patients and healthy controls; (C) Comparison of serum LTB_4 levels between PE patients and healthy controls. Results are expressed as means ± SEM (n=13 in each group). **$p<0.01$ and ***$p<0.001$ versus control group, two-tailed Student's t-test.
